# A New Strategy for Enhancing Postharvest Quality of Sweet Cherry: High-Voltage Electrostatic Field Improves the Physicochemical Properties and Fungal Community

**DOI:** 10.3390/foods13223670

**Published:** 2024-11-18

**Authors:** Yanlong Liu, Lulu Zhang, Tan Hu, Qiongyin Liu, Shuya Zhou, Yi Zhao, Abdul-Nabi Jatt, Caili Zhang, Hansheng Gong

**Affiliations:** 1 School of Food Engineering, Ludong University, Yantai 264025, China; liuyanlong314@163.com (Y.L.); 19712075353@163.com (L.Z.); liuqyabc123@163.com (Q.L.); zhoushuyarita@163.com (S.Z.); 15098978125@163.com (Y.Z.); hsgong_221@163.com (H.G.); 2 Yantai Key Laboratory of Nanoscience and Technology for Prepared Food, Yantai Engineering Research Center of Green Food Processing and Quality Control, Yantai 264025, China; 3 School of Materials Science and Engineering, Nanyang Technological University, Singapore 639798, Singapore; 4 Institute of Microbiology, University of Sindh, Jamshoro 76080, Pakistan; abdul.nabi@usindh.edu.pk

**Keywords:** sweet cherry, high-voltage electrostatic field, physicochemical properties, fungal community

## Abstract

Sweet cherry has a short shelf life due to the occurrence of senescence and fungal infection after harvest. This study aimed to study the effects of high-voltage electrostatic field (HVEF) on the physicochemical properties and fungal composition of sweet cherry during cold storage. The experiments were conducted at 4 °C for 28 days and the quality indicators were determined every 7 days during the period of storage. The fungal composition on sweet cherry was determined using high-throughput sequencing. The results showed that HVEF could better maintain the total soluble solids and inhibit the respiration of cherries. The decay incidence in sweet cherries was decreased by HVEF during cold storage. High-throughput sequencing revealed that HVEF could alter the fungal community and increase the fungal diversity on sweet cherries. Compared with the control group, HVEF decreased the abundance of *Alternaria* and *Cladosporium* on sweet cherries, while *Aureobasidium*, as a nonpathogenic fungus, increased and became the dominant strain at the end of the storage period. In summary, HVEF can improve the physicochemical properties of sweet cherry by inhibiting respiration and can reduce decay incidence by inhibiting specific pathogenic fungi. HVEF is expected to become an efficient and promising technology for the preservation of fruit.

## 1. Introduction

Sweet cherry (*Prunus avium* L.) is highly popular worldwide due to its flavor, taste, and nutrition [1]. Sweet cherry is rich in phenolics, anthocyanins, flavonoids, and ascorbic acid, and it has various physiological functions, such as antioxidant, anti-inflammatory, and antiobesity effects [2,3]. Due to vigorous postharvest respiration, sweet cherry is susceptible to aging, which causes changes in its texture and flavor, thereby affecting its sensory quality and nutritional value [4,5]. During the storage of sweet cherry, extensive fungal proliferation leads to fruit decay, resulting in serious economic losses [6,7]. Therefore, proper preservation methods are urgently required to maintain the quality and commercial value of sweet cherry.

Chemical preservation measures are widely used in the storage of sweet cherry in order to regulate its physiological metabolism or inhibit microbes [8,9]. A composite coating comprising *Eryngium campestre* essential oil wrapped in nanochitosan reduced the weight loss and total bacterial count, and increased the total phenolic content and antioxidant activity [10]. However, multiple chemical substances were used in the process of preparing the polymer coating, and this process was complex; it is, therefore, not suitable for continuous industrial production. Melatonin is able to delay the symptoms of aging by reducing respiration rate and maintaining a high TSS and TSS/TA ratio [11]. Natamycin can decrease decay incidence in sweet cherry by reducing yeast and mold counts and inhibiting pathogenic fungi such as *Alternaria* and *Cladosporium* [12]. However, chemical residues and complex processing procedures are not conducive to the application of chemical preservation. Due to consumers’ concerns about the safety and environmental pollution of chemical preservatives, physical preservation has attracted increasing attention [13]. Low temperature is the easiest way to maintain the quality of fruit. The skin of sweet cherry is particularly thin and easily infected by pathogenic fungi. Previous studies have shown that low temperature alone is not sufficient to completely suppress pathogenic fungi in sweet cherry [12,14]. For example, after 28 days of storage at 4 °C, the yeast and mold count of sweet cherry was increased by 2.3 log CFU/g [12]. In other studies, the decay incidence in sweet cherry was as high as 41% and 82.33%, respectively, after storage at 0 °C for 45 days and 63 days [4,9]. It has also been found that packaging using modified atmospheres could decrease the polygalacturonase activity and loss of elasticity in sweet cherry, but it has no significant effect on total soluble solids (TSS) [15]. As a result, it is necessary to explore efficient and safe techniques for the preservation of sweet cherry.

In recent years, the application of high-voltage electrostatic field (HVEF) in the preservation of fruit has garnered increasing interest [13,16,17]. HVEF preservation is a low-energy-consumption and chemical-free technology that enhances the maintenance of fruit and vegetable quality [18]. HVEF can inhibit the respiration of pineapple and pak choi, thereby delaying aging [19,20]. The TSS of cabbage and tomato were maintained at higher levels after HVEF treatment [21,22]. HVEF can better maintain the contents of ascorbic acid and total phenols in jujube and pomegranate, as well as the activities of antioxidant-related enzymes such as superoxide dismutase, catalase, and ascorbate peroxidase; this leads to an improvement in the antioxidant capacity of fruit [13,23]. Moreover, HVEF decreases the bacterial count in tomato and pak choi during storage [20,22]. The exact mechanism by which HVEF affects fruit quality is still under debate. The prevailing hypothesis is that HVEF ionizes the air to produce unstable ozone and air negative ions, which can decompose ethylene, reduce the stomatal opening of fruit, and inhibit microorganisms [13,20,23]. The application of HVEF in the preservation of fruit does not require the addition of chemicals, avoiding the production of chemical residues and environmental pollution. Meanwhile, HVEF treatment can be carried out directly in the refrigerator, offering a simple and timesaving method. However, the impact of HVEF on the postharvest qualities of sweet cherry remains unclear.

Microorganisms, including beneficial and pathogenic microorganisms, are an important component of fruit [24]. Plants have a highly diverse and dynamic microbial community, which is relevant to the health of the host [25,26]. The significant proliferation of pathogenic microorganisms can seriously disrupt microecological balance and reduce microbial diversity, ultimately leading to fruit decay [7]. Therefore, analyses of the composition and diversity of the fungal community of postharvest fruit could elucidate the interaction between the fungal community and the mechanism by which fruit diseases are controlled. However, previous studies have mainly focused on the effects of HVEF on the physicochemical quality, antioxidant capacity, and total bacterial count of fruits and vegetables [20,22,23], and the effects of fungal community and diversity have not attracted much attention. Until now, the effect of HVEF on the postharvest fungal community and diversity of sweet cherry has rarely been studied.

Therefore, the objective of this study was to analyze the effect of HVEF on the physicochemical properties and fungal diversity of sweet cherry under refrigeration in order to provide new insights into the preservation of sweet cherry.

## 2. Materials and Methods

### 2.1. Materials

Sweet cherries (cv. Tieton) were picked at commercial ripeness in Yantai, China (121°29′ E, 37°23′ N), and were immediately sent to the lab at Ludong University and precooled at 4 °C for 12 h. Sweet cherries with a uniform appearance and without disease or mechanical damage were selected for the experiment.

### 2.2. Sample Treatment

The fruit were randomly divided into two groups, named control and HVEF. The sweet cherries in the two groups were packed into polyethylene boxes (500 g/box). The samples in the control group were stored at 4 °C. The samples in the HVEF group were stored in a refrigerator equipped with an electrostatic field module (3 kV/cm, BMT-JDCJD200, Bomeite, Binzhou, China) at 4 °C. The physicochemical properties of the sweet cherries were measured every 7 days.

### 2.3. Determination of Physicochemical Properties

Cherry juice was obtained using five sweet cherries for the determination of the TSS and titratable acidity (TA). TSS was measured with a Brix meter (Atago PAL-1, Tokyo, Japan) [27]. TA was measured via titration and expressed as grams of malic acid/100 g of fresh sweet cherry [11]. The color of the fruit was determined with a CR400 colorimeter (Konica Minolta, Tokyo, Japan) and expressed as CIE color coordinates (L*, a*, b*) according to the method of Pholsin et al. [28].

### 2.4. Respiration Rate

The sweet cherries were placed in a 5 L airtight receptacle for 60 min. The CO_2_ concentration in the receptacle was measured using a T7001 gas detector (Telaire, Goleta, CA, USA) according to the method proposed by Zhong et al. [29]. The respiration rate was defined as the mass of CO_2_ released per kg fruit per hour.

### 2.5. Determination of Decay Incidence

The presence of visible fungal hyphae or soft rot on the sweet cherries was considered as decay. The decay incidence was defined as the ratio of the rotten fruit to the total amount of fruit [30].

### 2.6. Determination of Yeast and Mold Count

The sweet cherries were homogenized and diluted with sterile physiological saline in a 10-fold gradient. The dilutions (100 μL) were spread on potato dextrose agar (PDA) and incubated at 28 °C for 4 d. The colony counts on the PDA plates were used to calculate the yeast and mold count, which were expressed as log CFU/g.

### 2.7. DNA Extraction and Amplicon Sequencing

The microbial DNA from sweet cherries was extracted with hexadecyl trimethyl ammonium bromide and detected using 1% agarose gel electrophoresis. PCR amplification was performed for the ITS1-1F gene using a primer (5′-CTTGGTCATTTAGAGGAAGTAA-3′, 5′-GCTGCGTTCTTCATCGATGC-3′) [31]. The PCR amplicons were purified using a DNA purification kit (TianGen, Beijing, China). Sequencing libraries were constructed using purified amplicons and sequenced in the NovaSeq 6000 platform (San Diego, CA, USA). The fungal amplicon sequencing data have been uploaded to the NCBI database (BioProject ID: PRJNA1173949).

### 2.8. Bioinformatics Analysis

The original paired-end reads were combined using FLASH (http://ccb.jhu.edu/software/FLASH/, accessed on 16 July 2023) to obtain raw tags, which were then used to filter low-quality and low-length sequences to obtain qualified tags. The effective tags were further obtained by removing the chimera sequences. The effective tags were denoised using QIIME2 (https://qiime2.org, accessed on 16 July 2023) in order to obtain amplicon sequence variants (ASVs). The Unite database (https://unite.ut.ee, accessed on 16 July 2023) was used to annotate species. The FUNGuild tool (https://github.com/UMNFuN/FUNGuild, accessed on 16 July 2023) was used for fungal function annotation [32].

### 2.9. Statistical Analysis

The results are expressed as mean ± SD based on three replicates. SPSS 19.0 (SPSS, Chicago, IL, USA) was used to perform the independent T analysis test at *p* < 0.05. The alpha diversity indexes were analyzed using the Kruskal–Wallis test.

## 3. Results

### 3.1. Physicochemical Properties

**Figure 1:**
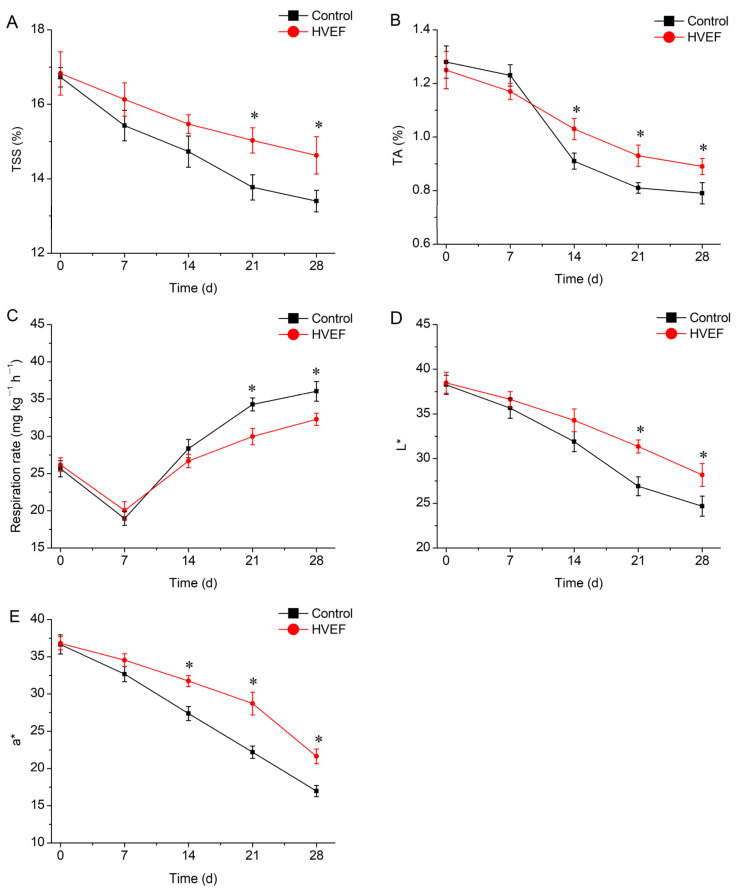
Effect of HVEF on physicochemical properties of sweet cherry. The TSS (**A**), TA (**B**), respiration rate (**C**), L* value (**D**), and a* value (**E**) of sweet cherry; * represents significant difference at *p* < 0.05 (*n* = 3).

As shown in Figure 1A, the TSS of sweet cherry in both the control and HVEF groups decreased during the storage period. The HVEF group showed significantly higher TSS values compared with the control group at days 21–28. The TA values of the HVEF group were significantly higher compared with the control group from day 14 to day 28 (Figure 1B). HVEF significantly decreased the respiration rate at days 21–28 (Figure 1C). The L* values continued to decrease during the storage period, and significantly higher L* values were observed in the HVEF group at days 21–28 (Figure 1D). Similar to the L* value, the HVEF group exhibited significantly higher a* values at days 14–28 (Figure 1E).

### 3.2. Decay Incidence

**Figure 2:**
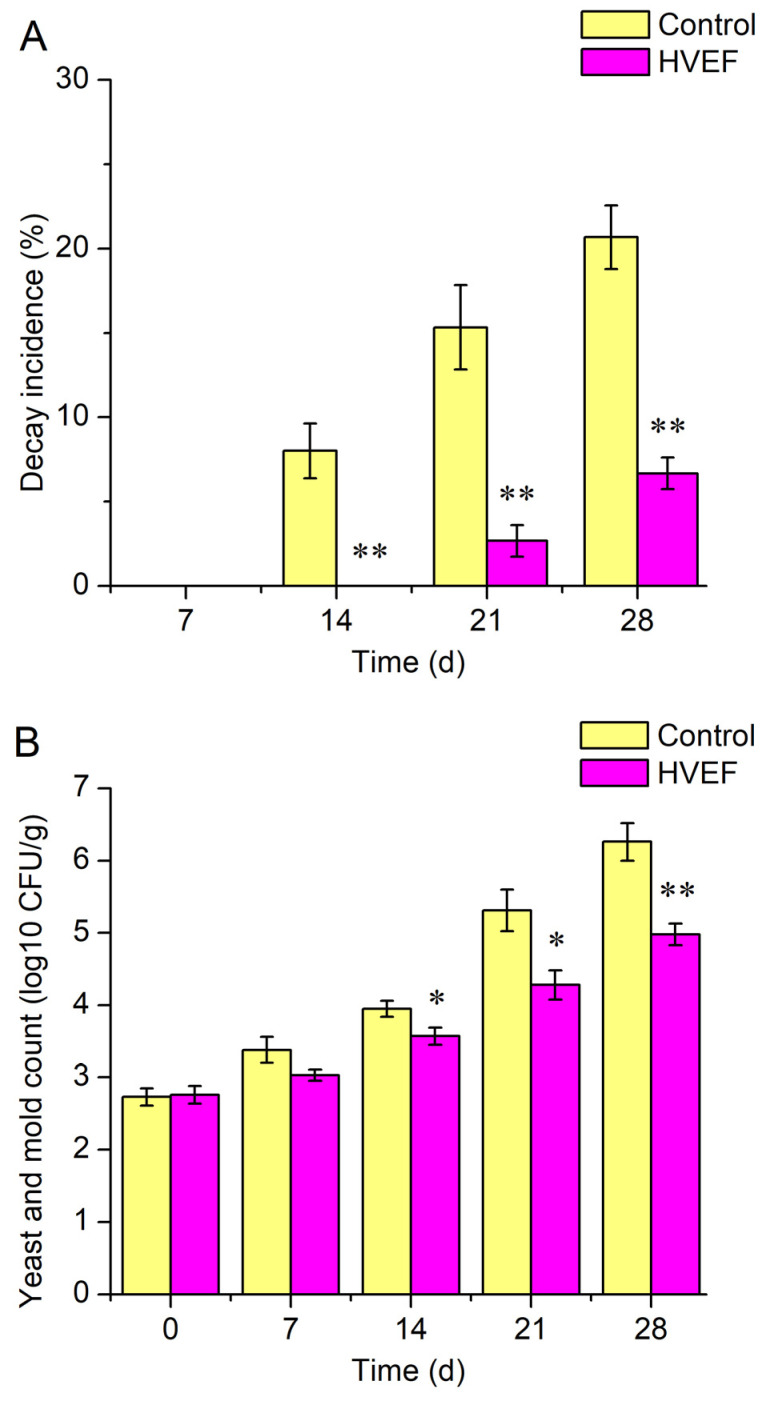
Effect of HVEF on decay incidence (**A**) and yeast and mold count (**B**) of sweet cherry; * and ** represent significant difference at *p* < 0.05 and *p* < 0.01, respectively (*n* = 3).

The control group and the HVEF group exhibited decay at day 14 and day 21, respectively (Figure 2A). The HVEF group showed significantly lower decay incidences at days 14–28 compared with the control group.

### 3.3. Yeast and Mold Count

The yeast and mold counts of both groups increased with storage time (Figure 2B). The yeast and mold counts of the HVEF group were significantly higher compared with the control group at days 14–28. At the end of the storage period, the yeast and mold count of the HVEF group was reduced by 1.28 log CFU/g compared with the control group.

### 3.4. Fungal Amplicon Sequencing Results

**Figure 3:**
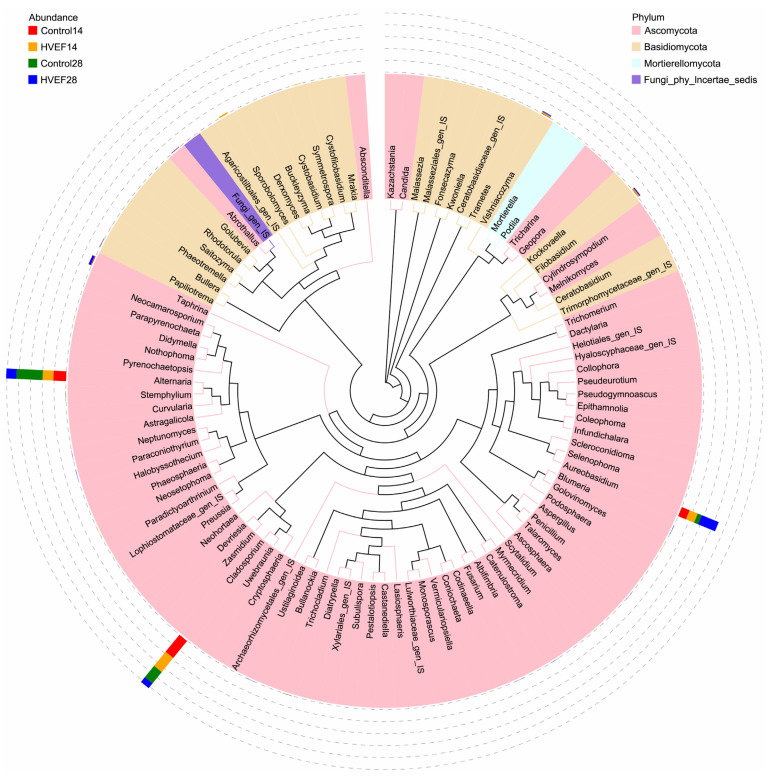
The phylogenetic tree of fungi on sweet cherry at genus level; 14 and 28 represent the days of storage, and IS represents incertae sedis.

The ITS sequencing data contained 1,088,367 original sequences, from which 1,043,371 effective tags were obtained (Table 1). The total effective tags accounted for 95.87% of the number of original paired-end sequences, indicating that the sequencing data were reliable. Finally, 329 ASVs were obtained from the effective tags. These ASVs were annotated as 5 phyla, 20 classes, 45 orders, 81 families, 104 genera, and 109 species. A phylogenetic tree was constructed based on the top 100 genera with relative abundance, which is shown in Figure 3. The genera of fungi present on sweet cherry mostly belonged to *Ascomycota* (72%), followed by *Basidiomycota* (25%). *Alternaria*, *Cladosporium,* and *Aureobasidium* were the dominant genera present on sweet cherry.

### 3.5. Fungal Diversity

**Figure 4:**
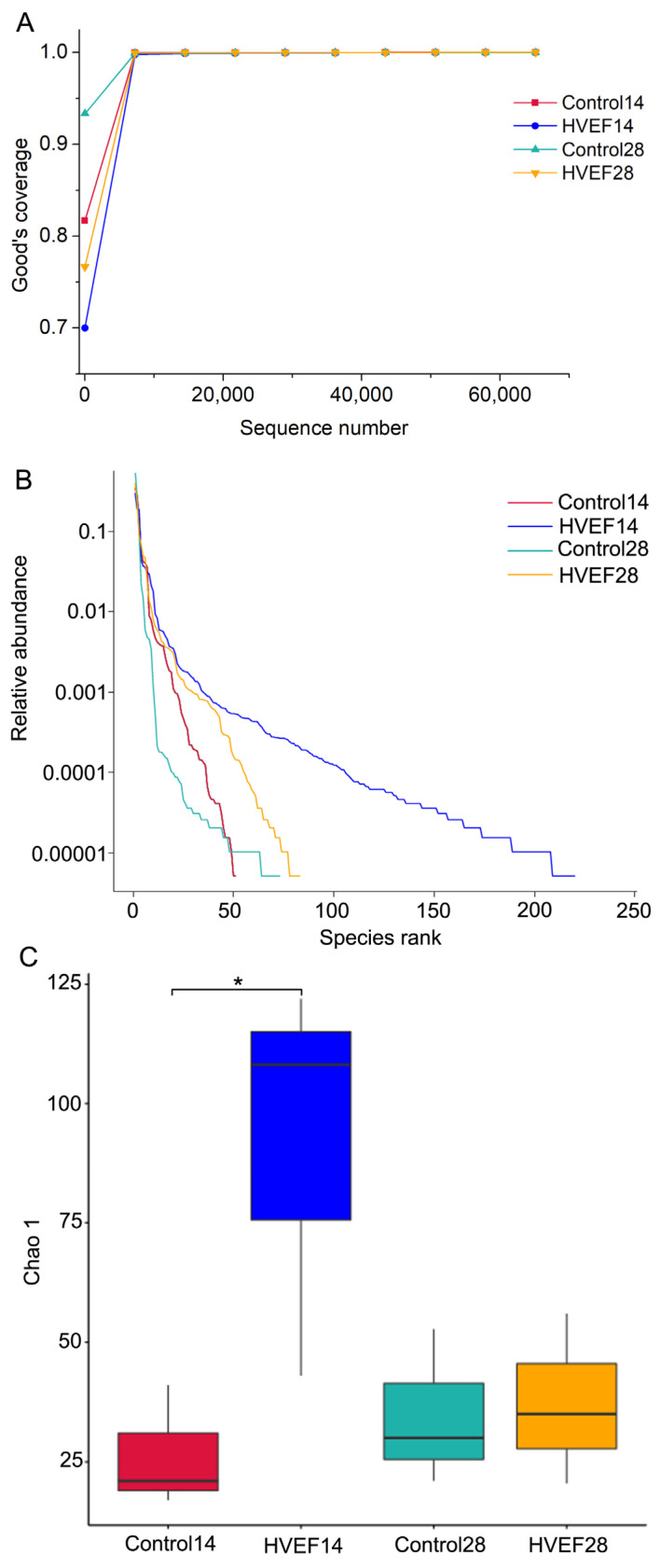
Effect of HVEF on fungal alpha diversity of sweet cherry. (**A**) Rarefaction curves; (**B**) rank abundance curves; (**C**) alpha diversity indexes; 14 and 28 represent the days of storage. The diversity indexes were analyzed by Kruskal–Wallis test; * represents significant difference (*p* < 0.05).

Good’s coverage is usually used to evaluate the likelihood that ITS amplicons can be sequenced [33]. The results showed that the Good’s coverage of all samples was 100% (Figure 4A). As shown in Figure 4B, the rank abundance curves of samples treated with HVEF at day 14 and day 28 were longer on the horizontal axis than those of the control group. The alpha diversity index was represented as Chao 1, which was used to reflect species richness [34]. The HVEF group showed a higher Chao 1 index compared with the control group at day 14 (Figure 4C), indicating that HVEF treatment can increase the fungal diversity of sweet cherry. There was no difference in the Chao 1 index between the two groups at day 28.

### 3.6. Fungal Community

**Figure 5:**
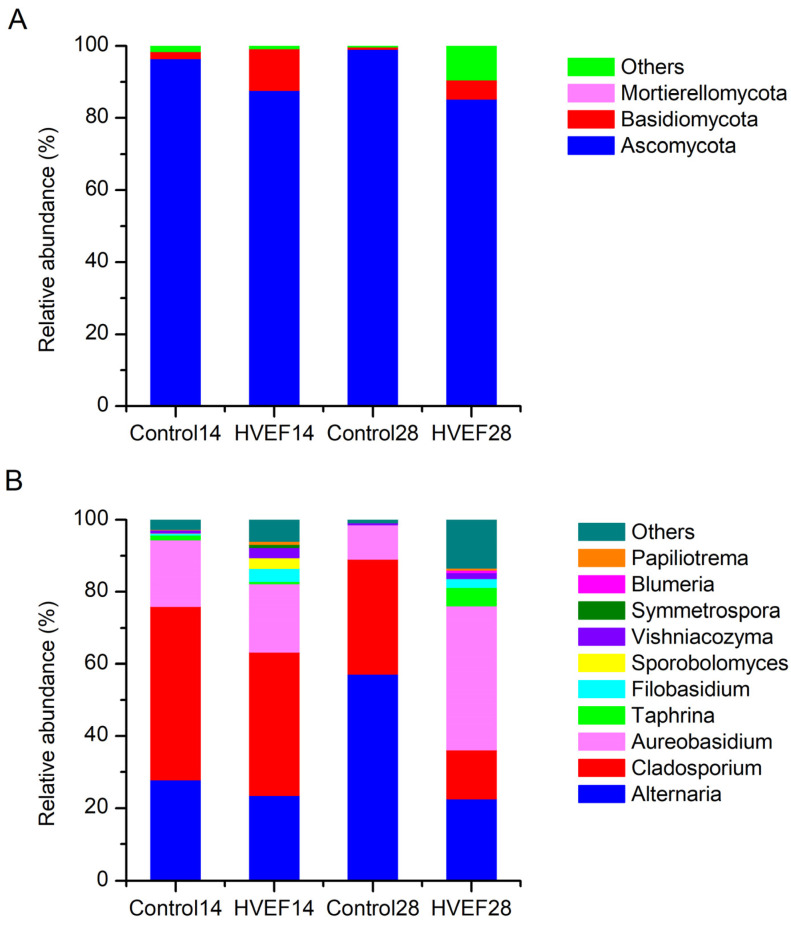
Fungal community composition on sweet cherry. (**A**) Fungal abundance at phylum level; (**B**) fungal abundance at genus level. Note: 14 and 28 represent the days of storage.

*Ascomycota* was the most important phylum of fungi on sweet cherry, with a relative abundance ranging from 85.38% to 99.25% (Figure 5A). HVEF decreased the abundance of *Ascomycota* and increased that of *Basidiomycota* at day 14 and day 28. As shown in Figure 5B, the dominant fungi in the control group at day 14 were *Cladosporium* (48.13%), *Alternaria* (27.85%), and *Aureobasidium* (18.53%). Compared with the control group, both *Cladosporium* (39.70%) and *Alternaria* (23.60%) in the HVEF group decreased. Furthermore, HVEF increased the abundance of nondominant fungi. At day 28, the abundance of *Alternaria* in the control group increased up to 57.23%, followed by *Cladosporium* (31.87%) and *Aureobasidium* (9.57%). HVEF reduced the abundance of *Cladosporium* to 13.60%, which was the lowest among all samples. *Alternaria* (22.54%) was decreased by 34.69% compared with the control group. *Cladosporium* and *Alternaria* are considered pathogenic fungi of sweet cherry [12]. The HVEF group had the lowest abundance of total pathogens (*Cladosporium* and *Alternaria*) at day 28.

### 3.7. Fungi Functional Annotation

**Figure 6:**
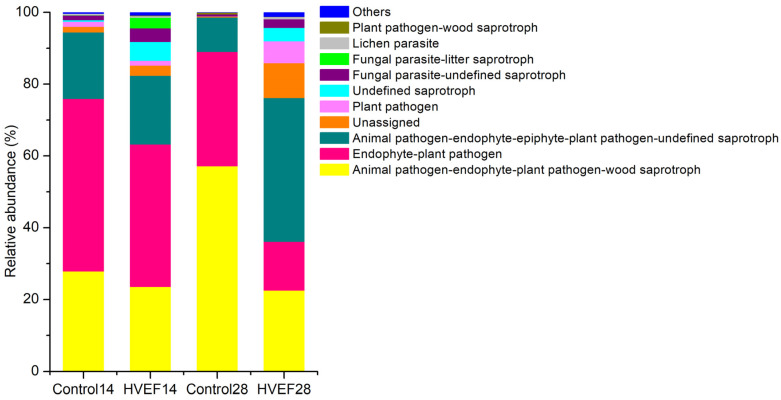
Relative abundance of fungal function (FUNGuild) annotation; 14 and 28 represent the days of storage.

After 14 days of storage, the abundance of animal pathogen–endophyte–plant pathogen–wood saprotroph and endophyte–plant pathogen in the HVEF group decreased (Figure 6). In the control group, the abundance of animal pathogen–endophyte–plant pathogen–wood saprotroph increased by 105.49% at day 28 compared with that at day 14. The abundance of animal pathogen–endophyte–plant pathogen–wood saprotroph and endophyte–plant pathogen in the HVEF group decreased sharply compared with the control group at day 28, while the abundance of animal pathogen–endophyte–epiphyte–plant pathogen–undefined saprotroph increased threefold. In this study, animal pathogen–endophyte–plant pathogen–wood saprotroph, endophyte–plant pathogen, and animal pathogen–endophyte–epiphyte–plant pathogen–undefined saprotroph represented *Alternaria*, *Cladosporium,* and *Aureobasidium*, respectively.

### 3.8. Correlation Analysis

**Figure 7:**
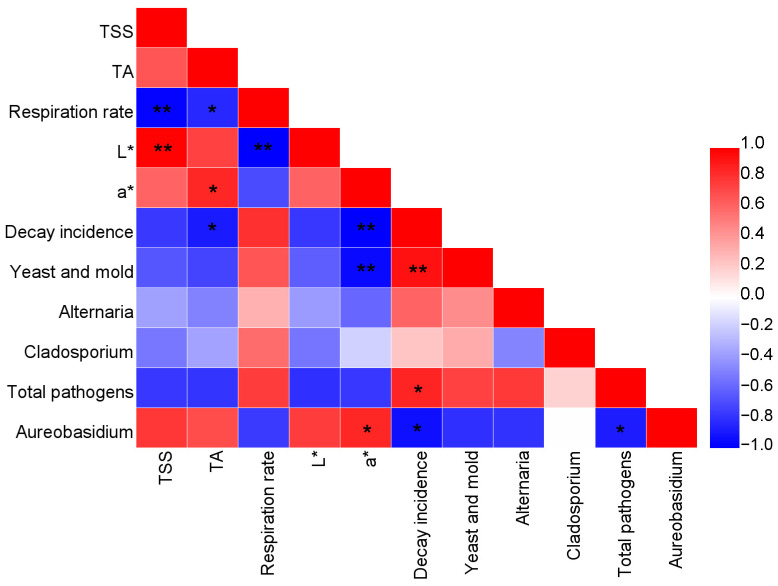
Correlation analysis of the quality parameters of sweet cherry; * and ** represent difference at *p* < 0.05 and *p* < 0.01, respectively.

The correlation analysis of the quality parameters of sweet cherry is shown in Figure 7. TSS was negatively correlated with the respiration rate and positively correlated with the L* value. TA showed a negative correlation with the respiration rate and decay incidence, and a positive correlation with the a* value. There was a negative correlation between the respiration rate and L* value. The a* value had a negative correlation with the decay incidence and yeast and mold count, and a positive correlation with the abundance of *Aureobasidium*. The decay incidence showed a positive correlation with the yeast and mold count and the abundance of total pathogens, and a negative correlation with the abundance of *Aureobasidium*. A negative correlation was found between the abundance of total pathogens and the abundance of *Aureobasidium*.

## 4. Discussion

HVEF is used for the preservation of fruit because it can improve their physiological quality [19,23]. Cheng et al. reported that low-voltage electrostatic fields could inhibit the decrease in total phenols in fresh-cut pineapples [16]. This study indicated that HVEF could contribute to the maintenance of TSS and TA, reducing the decay incidence and the yeast and mold count, and improving the fungal community and diversity of sweet cherry.

TSS is a key quality indicator for sweet cherry. The TSS of sweet cherry is affected by multiple factors during storage. Respiration usually reduces TSS due to the consumption of sugar, whereas polysaccharide hydrolysis increases TSS [14,35]. The respiration of sweet cherry was increased during storage [11,36], exhibiting a similar trend to that shown in this study. Therefore, the increased respiration of sweet cherry during storage might be responsible for the decrease in TSS over time. However, compared with the control, HVEF contributed to the maintenance of TSS in sweet cherry at days 21–28. Zhao et al. [22] found that HVEF delayed the reduction in TSS in cherry tomato. It was reported that the electrostatic field decreased the respiration of pineapple and persimmon [16,19], in accordance with our results. Exogenous HVEF could reduce epidermal stomatal opening and hinder sugar metabolism in fruits and vegetables [13], which supports the results of this study. Therefore, the ability of HVEF to maintain higher levels of TSS in sweet cherry might be attributed to the inhibition of respiration. In addition, the negative correlation between the TSS and respiration rate further confirms these results.

TA is a significant flavor parameter of fruit. Organic acids are substrates for fruit respiration and are reduced during cherry ripening [35]. HVEF reduced the TA of sweet cherry at days 14–28 compared with the control, which may be related to the decrease in the respiration rate. These results were supported by the negative correlation between TA and the respiration rate. It was reported that HVEF treatment inhibited the catabolism of organic acids during the storage of cherry tomato and maintained a lower pH [22], which was consistent with this study. Therefore, HVEF maintained a high TSS and TA by reducing respiratory metabolism, thus delaying aging and maintaining the high quality of sweet cherry.

Color is an important element that affects consumers’ preferences. In the CIE color coordinate system, L* and a* represent lightness and redness, respectively. In this study, HVEF delayed the decline in lightness at days 21–28 and the decline in redness at days 14–28, compared with the control, which prevented sweet cherry exhibiting a dark color. HVEF effectively maintained the lightness of cherry tomato and fresh-cut pineapple, in agreement with the results of this study [22,33]. The red color of sweet cherry is mainly attributed to anthocyanins [3]. During the ripening and storage of sweet cherry, the content of anthocyanins increases and the color darkens [37,38], which is consistent with the aging process in sweet cherry. Therefore, the lightness and redness of sweet cherry were better maintained in the HVEF group, which may be related to the reduction in anthocyanin synthesis. A previous study reported that HVEF reduced the anthocyanin content of pomegranate [23], which supported our results. HVEF improved the color of sweet cherry, which may be related to the inhibition of the aging process of sweet cherry by electrostatic field.

Decay incidence is an intuitive index used to assess the effect of storage on fruit. This study indicated that HVEF was able to reduce the decay incidence in sweet cherry at days 14–28 compared with the control group. It is generally believed that fungi are the main cause of the decay of sweet cherry [12]. Previous studies analyzed the number of fungi on fruit by determining the yeast and mold count to assess the risk of fruit decay and explain the cause of decay [14]. In this study, HVEF significantly decreased the yeast and mold counts at days 14–28 compared with the control group, which was consistent with the results regarding the decay incidence. Therefore, the lower decay incidence in the HVEF group may be related to the inhibition of fungal growth by HVEF. It has been shown that HVEF can decrease the microbial counts of tomato [22]. The total colony counts of pak choi were decreased as the electric field intensity increased [20]. These findings supported our results. The inhibition of microorganisms by HVEF may be related to the ozone produced by the ionization of air under electrostatic field conditions [22]. The ozone and ion mist generated by HVEF cause the denaturation of microbial protein and physiological metabolic disorders. In addition, HVEF can increase the membrane potential of cell membranes, causing the surface of the cell membrane to be punctured and leading to the leakage of intracellular contents [20]. However, there are few reports on the effect of HVEF on the composition of the fungal community, especially pathogenic fungi, during fruit storage. The mechanism via which HVEF decreases the incidence of decay could be explained by analyzing the pathogenic fungi through amplicon sequencing.

Plants have high levels of microbial diversity and dynamic microbial colonies, which are closely related to plant health [26]. Studies have increasingly used fungal diversity to analyze the relationship between fungi and fruit decay [24,26,39]. Therefore, this study characterized the fungal composition of sweet cherry using high-throughput sequencing. As the amount of sequencing increased, all the rarefaction curves ultimately became saturated, indicating that increasing the quantity of sequencing data would not affect fungal species [33]. Therefore, the sequencing depth of this study was sufficient and the sequencing results were reliable, enabling their use in the analysis of fungal community and diversity.

In this study, the rank abundance curve and alpha diversity index were used to evaluate the fungal diversity of sweet cherry. The rank abundance curve reflects the richness and evenness of microorganisms, and a larger span on the horizontal axis indicates a higher richness of microorganisms. The HVEF group had a larger span on the horizontal axis compared with the control group at day 14, indicating that HVEF treatment increased the richness of fungi on sweet cherry. The diversity index also signified that HVEF increased the fungal diversity on sweet cherry after 14 days of storage. At the same time, the HVEF group exhibited a lower decay incidence, which was in accordance with the results for diversity. Previous studies have indicated that a higher decay incidence usually corresponds to lower fungal alpha diversity [7,24], which is in agreement with the results of this study. The rank abundance curve indicated that the fungal richness of the HVEF group was higher compared with the control group after 28 days of storage, whereas there was no difference in the alpha diversity index; this indicates that different diversity assessment indicators might differ due to different perspectives. In other studies, although the treatment group had a lower decay incidence after storage, the fungal alpha diversity index was not significantly different between the treatment and control groups [40]. This phenomenon might be due to nonpathogenic fungi becoming dominant strains at the end of storage, such as *Aureobasidium* in this study, thereby reducing fungal diversity in the treatment group. This phenomenon suggested that fungal diversity was not the only indicator able to explain the decay incidence in fruit, and that it might need to be evaluated further. On the whole, HVEF was able to improve the fungal diversity on sweet cherry during cold storage.

Analyzing the changes in the type and relative abundance of fungi during fruit storage can explain the process of decay, providing important insights regarding the storage of fruit. Compared with the control, HVEF alters the fungal community composition on sweet cherry at the phylum and genus levels. Some specific pathogenic microorganisms become the dominant strains during the storage of fruit, which eventually leads to the decay of fruit. A previous study reported that the pathogenic fungi present on sweet cherry mainly include *Alternaria*, *Cladosporium*, *Botrytis,* and *Rhizopus* [41]. In this study, *Alternaria* and *Cladosporium* were the dominant fungi on sweet cherry and were most likely responsible for the decay of sweet cherry; these results agreed with previous reports. In addition, *Alternaria* and *Cladosporium* are unpleasant pathogens that reside on tomato, grape, jujube, etc. [40,42,43,44]. After 14 days of storage, HVEF inhibited *Alternaria* and *Cladosporium*, implying that HVEF may decrease the decay incidence in sweet cherry via the inhibition of specific pathogenic fungi. The abundance of total pathogens in the control group at day 28 was higher than that at day 14, indicating that the dominance of pathogenic fungi was expanded; this was in agreement with the higher decay incidence. The increase in pathogenic microorganisms can lead to the disruption of microbial ecological balance, thereby increasing the decay incidence in fruit [7,45]. *Cladosporium* was further inhibited by HVEF at day 28, indicating that HVEF had a good inhibitory effect on *Cladosporium*. The total abundance of *Alternaria* and *Cladosporium* in the HVEF group was also reduced, which agreed with the lower decay incidence observed. The correlation analysis showed that the decay incidence was positively correlated with the abundance of total pathogens, which supported these results. In addition, *Aureobasidium* was negatively correlated with total pathogens, suggesting that there might be a competitive relationship between *Aureobasidium* and pathogens. Therefore, it could be concluded that HVEF decreased the decay incidence in sweet cherry by inhibiting specific pathogenic fungi such *Alternaria* and *Cladosporium*.

FUNGuild can classify sequence pools into ecologically significant categories, and is widely used to analyze the function of fungal community [32]. Fungal functional analysis showed that the animal pathogen–endophyte–plant pathogen–wood saprotroph guild, represented by *Alternaria*, and the endophyte–plant pathogen guild, represented by *Cladosporium*, dominated in the control group. The HVEF treatment decreased the abundance of these two guilds. In particular, the total abundance of these two guilds in the HVEF group was only 36.14% at day 28, which was decreased by 59.44% compared with the control group. The results of FUNGuild were in line with the relative abundance of fungi. The functional analysis of fungi further indicated that HVEF could inhibit *Alternaria* and *Cladosporium* on sweet cherry, which led to a decrease in the decay incidence during storage.

The application of HVEF in the preservation of fruits and vegetables is still in the research stage. Due to the significant differences in the physiological characteristics of various fruits and vegetables, HVEF has different effects on various fruits and vegetables. For example, HVEF treatment had no effect on the electrolyte leakage of baby corn during storage [21] but increased the electrolyte leakage of tomato [23]. Overall, HVEF treatment can better maintain the quality of sweet cherry, pak choi [20], jujube [13], tomato [22], etc., during storage. In addition, HVEF treatment also had negative effects on some fruits; for example, it increased weight loss in pomegranate. In addition, the limitations of high costs, technological immaturity, and nonuniversal process parameters have not been fully addressed [22], which limits the application of HVEF in individual homes. Therefore, before HVEF can be widely applied globally, researchers must perform extensive and in-depth research on its impact on the qualities and physiological metabolism of various fruits and vegetables.

## 5. Conclusions

This study confirmed the effectiveness of employing HVEF treatment to improve the physicochemical properties of sweet cherry and regulate fungal composition and diversity during cold storage. Specifically, HVEF treatment had significant effects on the physicochemical properties of sweet cherry, such as inhibiting the decrease in TSS and TA, reducing the respiration rate, and maintaining higher L* and a* values. In addition, HVEF reduced the decay incidence and yeast and mold counts of sweet cherry. High-throughput sequencing showed that HVEF increased the fungal diversity on sweet cherry. HVEF also altered the fungal community on sweet cherry at both the phylum and genus levels. In particular, HVEF decreased the abundance of pathogenic fungi such as *Alternaria* and *Cladosporium*, while increasing the abundance of *Aureobasidium*, which contributed to a reduction in postharvest decay. The Pearson correlation analysis showed that the TSS, TA, and L^*^ values were closely related to the respiration rate. The decay incidence was closely related to the yeast and mold counts, the abundance of pathogens, and the abundance of *Aureobasidium*. In summary, HVEF contributed to improvement in the physicochemical properties and fungal composition of sweet cherry during refrigeration. HVEF could, therefore, be employed as a strategy for the maintenance of the postharvest quality of fruit.

## Figures and Tables

**Table 1:** Number of amplicon subsequences during data processing.

Sample	Paired-End Reads	Raw Tags	Qualified Tags	Effective Tags
Control14-1 *	92,724	87,065	86,885	85,477
Control14-2	92,824	92,521	92,472	91,699
Control14-3	85,869	85,590	85,548	84,830
HVEF14-1	67,848	66,885	66,828	66,108
HVEF14-2	98,985	94,188	94,089	91,959
HVEF14-3	100,094	94,469	94,427	93,864
Control28-1	89,719	89,441	89,398	88,534
Control28-2	94,810	94,356	94,315	93,544
Control28-3	91,571	91,307	91,273	90,882
HVEF28-1	88,532	88,159	88,078	87,374
HVEF28-2	87,645	84,399	84,334	83,614
HVEF28-3	97,746	86,314	86,240	85,486
Total	1,088,367	1,054,694	1,053,887	1,043,371

* 14 and 28 represent the days of storage, and the numbers after the days represent the parallel number.

## Data Availability

The data presented in this study are available on request from the corresponding author due to privacy.
